# The Significance of Matrix Metalloproteinases in Parasitic Infections Involving the Central Nervous System

**DOI:** 10.3390/pathogens2010105

**Published:** 2013-02-19

**Authors:** Fabrizio Bruschi, Barbara Pinto

**Affiliations:** Department of Translational Research, N.T.M.S., University of Pisa, School of Medicine, Via Roma, 55, 56126, Italy; E-Mail: barbara.pinto@dps.unipi.it (B.P.)

**Keywords:** matrix metalloproteinases, tissue inhibitor of metalloproteinases, malaria, trypanosomosis, toxoplasmosis, neurocysticercosis, angiostrongyloidosis

## Abstract

Matrix metalloproteinases (MMPs) represent a large family of over twenty different secreted or membrane-bound endopeptidases, involved in many physiological (embryogenesis, precursor or stem cell mobilization, tissue remodeling during wound healing, *etc.*), as well as pathological (inflammation, tumor progression and metastasis in cancer, vascular pathology, *etc*.) conditions. For a long time, MMPs were considered only for the ability to degrade extracellular matrix (ECM) molecules (e.g., collagen, laminin, fibronectin) and to release hidden epitopes from the ECM. In the last few years, it has been fully elucidated that these molecules have many other functions, mainly related to the immune response, in consideration of their effects on cytokines, hormones and chemokines. Among others, MMP-2 and MMP-9 are endopeptidases of the MMP family produced by neutrophils, macrophages and monocytes. When infection is associated with leukocyte influx into specific organs, immunopathology and collateral tissue damage may occur. In this review, the involvement of MMPs and, in particular, of gelatinases in both protozoan and helminth infections will be described. In cerebral malaria, for example, MMPs play a role in the pathogenesis of such diseases. Also, trypanosomosis and toxoplasmosis will be considered for protozoan infections, as well as neurocysticercosis and angiostrongyloidosis, as regards helminthiases. All these situations have in common the proteolytic action on the blood brain barrier, mediated by MMPs.

## 1. Introduction

Matrix metalloproteinases (MMPs) are a family of multi-domain Ca^2+^-dependent and Zn^2+^-containing endopeptidases, strictly related, which can degrade almost all components of the extracellular matrix (ECM), but also non-matrix proteins [1].

The aim of this review is to focus the attention on the role played by these enzymes in the pathogenesis of neurological involvement by parasitic infections, either caused by protozoa or helminths, with the intent to show that many mechanisms are common to the different situations. 

Before reviewing the data, which are accumulating in the literature, in these last few years, regarding different parasitic infections, we briefly discuss the structure and function of the main target of the endopeptidases, the matrix and then describe the different MMPs.

## 2. The matrix

The ECM is a complex structural entity surrounding and supporting cells that are found within mammalian tissues. The ECM has two basic forms, the interstitial extracellular matrix (ECM) and epithelial-cell associated basement membrane (BM) [2,3]. In most tissues, it is composed of several different macromolecules, mainly proteins mixed with fibrous glycoproteins, like structural proteins/glycoproteins (collagen and elastin), specialized proteins/glycoproteins, (fibrillin, fibronectin and laminin) and proteoglycans [4,5]. These are composed of a protein core to which long chains of repeating disaccharide units, termed glycosaminoglycans (GAGs), are attached.

ECM provides mechanical strength and protection and plays a role in the regulation of intercellular communication under normal and pathological conditions [6], such as apoptosis, angiogenesis and cell differentiation [7]. Most importantly, the ECM provides cell-matrix and tissue cohesion through adhesion proteins, which regulate cell functions that are vital for wound healing [3,8]. The ECM is also responsible for transmitting extracellular signals to cells and, ultimately, regulates cell proliferation, differentiation and death [9,10].

The amount of ECM varies in the different tissues, being scarce in the nervous and muscle tissue and, instead, abundant in cartilage and bone [11] and in blood [12]. Components of the ECM are produced inside the cell by resident cells [fibroblasts, chondrocytes, osteoblasts] and secreted into the ECM via exocytosis [13]. Once secreted, they then aggregate with the existing matrix. Differently from other tissues, the ECM in the Central Nervous System (CNS) lacks fibrillar proteins in physiological conditions. Instead, the brain ECM is rich in glycoproteins and proteoglycans [14]. It has been estimated that this ECM makes up about 20% of the CNS parenchyma [15]. 

Degradation of ECM is an important feature of development, morphogenesis, tissue repair and remodeling [16,17]. Proteolysis is a major process leading to changes in the ECM [16]. Several types of proteolytic enzymes are involved in ECM degradation. Indeed, the proteolytic system operating in human tissues is extremely complex, and more than 500 genes coding for proteases or protease-like proteins are present in the human genome. In this group, the major enzymes degrading the ECM are serine-proteases, cysteine-proteases and members of the family of matrix metalloproteinases (MMPs) [18].

## 3. The Role of MMPs in Normal and Pathological Conditions

MMPs participate in remodeling and degradation of ECM and basement membranes [BM], which occur throughout life in a number of physiological processes, during embryogenesis, cell proliferation, migration and differentiation, ovulation, mammary gland involution and proteolytic activation of growth factor, as well as in epidermal wound healing and tissue repair in response to injury (e.g., after myocardic infarction) [19,20,21]. 

Recent evidence has implicated MMPs in the regulation of other functions, including cell survival, angiogenesis, inflammation and signaling [22,23], as well as in neuronal physiology and plasticity of the adult brain [24]. Upregulation of MMP expression and activity is implicated in a number of acute and chronic pathological conditions, such as arthritis [25,26,27], cardiovascular disease [28,29,30], including acute myocardial infarction [28,30], chronic heart failure [28,30], chronic obstructive pulmonary disease [31,32,33], inflammatory bowel disease [22,34,35], diabetes [36] and tumor growth and metastasis [37,38,39,40,41].

The mature CNS normally contains non-detectable or low levels of most MMPs [23], but several become upregulated in neurological diseases, such as gliomas [42], viral infections, neuroinflammation [23], multiple sclerosis [34,43], Alzheimer’s disease [44], Guillain-Barré syndrome [45], amyotrophic lateral sclerosis [46], brain trauma and ischemia [47,48] and HIV-associated neurological disease [49].

### 3.1. The MMP Family

The MMP family currently comprises a group of at least 25 related, but distinct, soluble and membrane-bound enzymes, of which 24 are found in mammals [35]. They are synthesized by a wide range of cell types, mainly inflammatory cells, and are secreted to the extracellular space in an inactive form, called zymogen or pro-MMP [50]. Different cell types are capable of producing, for example, MMP-9, like monocytes [51], T-cells [52], neutrophils [53], astrocytes [54], microglia [55], eosinophils [56], macrophages [57] and endothelial cells [58].

Enzymes of the MMP family are able to degrade *in vitro* all ECM components. They are commonly divided into at least six subgroups (superfamilies) based on their substrate specificity and their amino acid sequence similarity (Table 1) [17,19].

**Table 1:** Matrix metalloproteinase (MMP) family subgroups.

Common name	MMP	Chromosomal location (human)	M.W. (kDa)	Collagen substrates	Some additional substrates*
*Collagenases*					
Collagenase-1	MMP-1	11q22-q23	55/45	I, II,III, VII, VIII, X,	Aggrecan, gelatin
Collagenase-2	MMP-8	11q21-q22	75/58	I, II, III, VII, VIII X	Aggrecan, gelatin, fibronectin
Collagenase-3	MP-13	11q22.3	60/48	I, II, III, IV, IX, X, XIV	Aggrecan, gelatin, fibronectin
Collagenase-4	MMP-18	( *Xenopus*)	70/53		
*Gelatinases*					
Gelatinasi A	MMP-2	16q13	72/66	I, II, III, IV, VII, X	Gelatin, fibronectin, fibrillin
Gelatinasi B	MMP-9	20q11.2-q13.1	92/86	IV, V	Gelatin, elastin, fibrillin
*Stromelysins*					
Stromelysin -1	MMP-3	11q23	57/45	II, III, IV,V,IX, X, XI	Gelatin, plasminogen
Stromelysin -2	MMP-10	11q22.3-q23	57/44	IV,	Laminin, fibronectin elastin,
Stromelysin -3	MMP-11	22q11.2	51/44	IV	Fibronectin, laminin, aggrecan
*Matrilysins*					
Matrylisin-1	MMP-7	11q21-q22	28/19	IV	Fibronectin, laminin, gelatin
Matrylisin-2	MMP-26	11p-15	28/19	IV	Fibrinogen, fibronectin, gelatin
Metalloelastase	MMP-12	11q22.2-q22.3	54/45	IV	Elastin, fibronectin, latent TNF
*MT-MMP*					
Tm-type I					
MT1-MMP	MMP-14	14q11-q12	66/56	I, II, III	Gelatin, fibronectin, laminin
MT2-MMP	MMP-15	15q13-q21	72/60		Gelatin, fibronectin, laminin
MT3-MMP	MMP-16	8q21	64/52	III	Gelatin, fibronectin, laminin
MT5-MMP	MMP-24	20q11.2	-/52		Gelatin, fibronectin, laminin
GPI-anchored					Fibrinogen, fibrin
MT4-MMP	MMP-17	12q24.3	57/63		Fibrin, gelatin
MT6-MMP	MMP-25	16p13.3		IV	Fibronectin, gelatin, laminin
*Other MMPs*					
	MMP-19	12q14	54/45	IV	
Enamelysin	MMP-20	11q22.3	54/22		Aggrecan, elastin, fibrillin Gelatin
	MMP-21	ND	70/53		Aggrecan
CA-MMP	MMP-23	1p36.3			Aggrecan
	MMP-27	11q24			Gelatin, casein, fibronectin
Epylisin	MMP-28	17q21.1	56/45		Casein

ND = not determined. TNF = tumor necrosis factor. *The list of substrates is by no means exhaustive.

**Figure 1:**
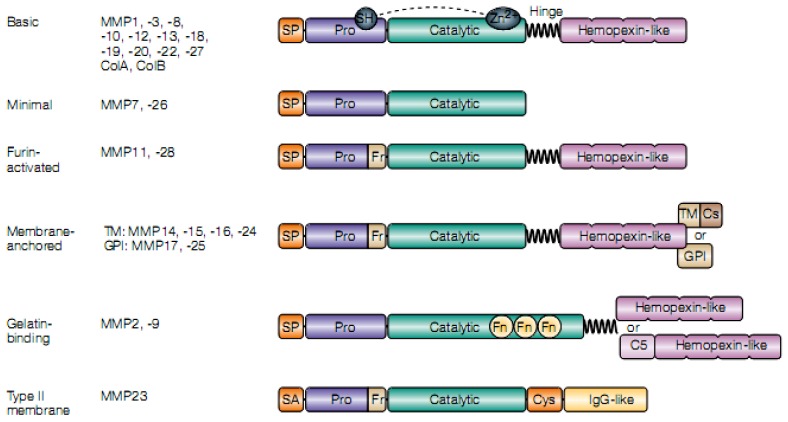
Domain structure of the mammalian MMP family. The important features of matrix metalloproteinases (MMPs) are illustrated, showing the minimal domain structures. Although MMPs are often subdivided into groups on the basis of differences in domain composition (shown here), there is little consensus in the field about how such subdivisions should be assigned. Domain structure alone does not predict function. One clear division is between MMPs that are secreted and those that are anchored to the cell surface by an intrinsic motif: namely, a transmembrane (TM) domain (MMP14, -15, -16 and -24), a glycosylphosphatidylinositol (GPI) anchor (MMP17 and MMP25) or an amino (N)-terminal signal anchor (SA) (MMP23). Both the TM domains and GPI anchors are attached to the hemopexin-like domain by a short linker. As discussed in the text, the secreted MMPs might still be confined to the cell surface through interactions with specific accessory macromolecules. Because the mechanisms that control activation (that is, conversion of proMMP to active MMP) are key steps in the regulation of proteolysis, another grouping of the MMPs can be made on the basis of intracellular activation by furin proteinases. Nine MMPs, including all of the membrane-anchored enzymes, have a furin-recognition domain. C5, type-V-collagen-like domain; Col, collagenase-like protein; Cs, cytosolic; Cys, cysteine array; Fn, fibronectin repeat; Fr, furin-cleavage site; Pro, pro-domain; SH, thiol group; SP, signal peptide; Zn, zinc. From [35], with permission.

All members of this family share a basic structure consisting of some common functional domains *i.e*., a signal peptide, a pro-peptide and a catalytic domain, containing a Zn^2+^ binding site (Figure 1). 

Matrilysins 1 and 2 (MMP-7, MMP-26) are the smaller MMPs actually known [59,60,61].

With the exception of MMP-7, -23 and -26, MMPs have a proline-rich hinge region and a carboxy (C-)terminal hemopexin-like domain, which is involved in substrate recognition [35,62,63]. However, some MMPs have additional domains, such as a C5 (Type-V-collagen-like) domain and transmembrane or a cytoplasmic domain [62,64]. 

Collagenases are generally able to cleave the interstitial collagens I, II and III and to digest other ECM, as well as non-ECM proteins [1,65]. 

The gelatinase group, which consists of MMP-2 and MMP-9, mainly digests gelatin, the denatured form of collagen [1,65].

The stromelysins MMP-3 and MMP-10 digest ECM components, such as collagen IV and fibronectin. MMP-11 is also called stromelysin-3, but its sequence and substrate specificity are different from that of MMP-3 and MMP-10. Some authors place MMP-11 in the heterogeneous subgroup [1,65]. 

Human macrophage elastase (MMP-12), another member of the stromelysin subgroup, was initially found in alveolar macrophages of cigarette smokers [66]. 

Matrilysins are categorized differently into the MMP groups by some authors [1,20,35,67]. Matrilysins digest several ECM components, such as fibronectin and gelatin [68,69,70]. They lack the C-terminal hemopexin-like domain present in all other MMPs and are therefore also called the “minimal-domain MMPs” [71,72,73]. 

The membrane-type matrix metalloproteinases (MT-MMP), of which six forms are known, can digest a number of ECM proteins, such as gelatin, fibronectin and laminin [74]. While most of the MMPs are secreted, the MT-MMPs are membrane-associated, and a number of these have cytoplasmic domains, which may be important in cellular signaling [75]. MT-MMPs in mammals include four type-I transmembrane proteins (MT1-, MT-2, MT-3, MT-5-MMP) and two glycophosphatidylinositol-anchored proteins (MT4-MMP and MT6-MMP) [20]. Moreover, most MT-MMPs can activate pro-MMP-2 [1,65,76]. In addition to the highly conserved MMP functional domains, the MT-MMPs have additional insertion sequences (IS) that confer unique functional roles [75]. 

The remaining MMPs are pooled in a heterogeneous subgroup, because of their different substrate specificity, amino acid sequence or domain organization [1,65,77]. This group includes MMP-19, MMP-20, MMP-21, MMP-23, MMP-27 and MMP-28, which can cleave substrates, such as elastin and aggrecan [1,65,77,78].

Regulation of MMPs has been recently nicely focused by several authors [17,19,23,24,35,50]. *In vivo* activity of MMPs is controlled at multiple levels. Under physiological conditions, the expression of many MMPs is precisely regulated at the level of transcription, which represents the major level of MMP regulation [79]. In normal tissues, MMP expression is constitutively low, but their synthesis is rapidly induced during tissue remodeling. Transcription of MMPs is regulated either positively or negatively by various effectors, including growth factors and cytokines, such as interleukins (IL-1, IL-4 and IL-6), chemokines, transforming growth factors (EGF, HGF and TGFß) or tumor necrosis factor alpha (TNFα) and other factors [35,50,76,80,81,82,83].

Post-translational modifications, such as activation of pro-MMP precursor zymogens and acetylation [83], provide another level of MMP regulation. In fact, most MMPs are generally synthesized by cells in a latent form as pre-pro-enzymes and activated extracellularly [19]. The signal peptide is removed during translation and pro-MMPs are generated [17]. Activation of zymogens represents an essential regulatory step of MMP activation and activity. Latency of the pro-MMPs is maintained by the interaction between the thiol group of a conserved cysteine residue (Cys^73^) in the prodomain and the Zn^2+ ^of the catalytic site [35,84]. They are converted to active proteinases by disruption of this interaction, a process known as the cysteine-switch mechanism [85], which can be achieved by proteolysis of the pro-domain or by modification of the cysteine thiol group [35]. Glycosylation may provide an additional level of regulation [86]. 

The extracellular proteolytic activation of the pro-enzyme is controlled by several steps involving other MMPs and serine proteinases, such as plasmin [50,76,81]. 

Upon activation, MMPs are further regulated by endogenous inhibitors, autodegradation and selective endocytosis. For instance, MMP-2, 9 and 13 are internalized through a low density lipoprotein receptor-related protein (LRP) mechanism [87]. Control over MMP activity may involve specific endogenous inhibitors, such as α2-macroglobulin, and tissue inhibitors of MMPs (TIMPs), as stated by Yong *et al*. [23] and Parks *et al*. [35]. 

TIMPS are a family of secretory proteins that are able to inhibit MMP activity in the extracellular environment in a 1:1 molar stoichiometry [19]. Four TIMPS have been identified at a gene level in mammals, namely TIMP-1, -2, -3 and -4 [88]. In normal cellular environment, TIMPs strictly regulate MMP activity [17,19,89]. TIMPS are expressed in various tissues and by many cell types, and their expression is regulated during development and tissue remodeling [89]. TIMPS inhibit almost all MMPs tested. However, they differ in their affinity for specific MMPs, and their action does not always lead to inhibition [35]. Their amino-terminal domain is crucial for the inhibitory activity, and it binds to the active site of the MMPs [17,88], while the C-terminal domain interacts with the hemopexin domain of MMP proenzymes [20]. The balance between MMPs and their TIMPs is a critical factor in normal and pathological tissue remodeling [17,89,90]. 

TIMPS show a high level of heterogeneity, suggesting MMP-independent functions [89]. Indeed, some TIMPS also have anti-angiogenetic [91,92] and anti-apoptotic effects [93,94].

An additional control of MMPs regulation is provided by factors, such as substrate availability and affinity, as well as compartmentalization. Reviews of this arguments have been recently provided by some authors [35,95].

## 4. Matrix Metalloproteinase Biology in Parasitic Infections of CNS

### 4.1. MMPs and Protozoan Infections

We will give now an overview of the state-of-the-art about the role of MMPs and TIMPs in parasitic infections, either caused by protozoa or by helminths, responsible for pathology at the CNS level, focusing mainly on *in vivo* results in humans, as well as in experimental models when appropriate to compare with the human pathology, referring the reader to more general reviews on this topic [96].

#### 4.1.1. Malaria

Malaria is one of major public health problems at a global level, causing between 300 and 500 million clinical cases and about 1 million deaths each year. In humans, one of five *Plasmodium* spp. is the etiological agent, but *Plasmodium falciparum* represents the most dangerous one, especially in Sub-Saharan Africa [97,98]. One of the most important causes of fatal malaria is represented by cerebral malaria (CM), which is the result of accumulation, as well as adhesion, to endothelial cells of parasitized red blood cells in the capillaries and post-capillary venules of the brain with the following hypoxia [99]. Pro-inflammatory cytokines, such as tumor necrosis factor (TNF), interleukin (IL)-1 and -6, elicited by the immune response against the parasite, play an important role in upregulating adhesion molecules on the endothelial cell surface and then aggravating the red blood cell sequestration [100]. Cell adhesion molecules expression and TNF-α and IL-1β levels have been observed to be increased in postmortem brains of children with CM, especially in the cerebellum [101]. 

The parasite escapes the host immune response with different strategies, one of which is represented by the coding and production of variant proteins present at the surface level, such as, for example, the *P. falciparum* erythrocyte membrane protein-1 (PfEMP-1), which allows the parasite to bind to different molecules present on the endothelial cell surface, facilitating its sequestration in the blood capillaries [100]. These sophisticated escape mechanisms into various organs cause the mechanical blockage of blood vessels and local inflammation, leading to organ-specific disease syndromes, such as placental malaria and CM [100,102].

Different barrier layers limit and regulate molecular exchange at the interfaces between the blood and the neural tissue or its fluid spaces. The blood brain barrier (BBB), formed by the cerebrovascular endothelial cells between blood and brain interstitial fluid, is a selectively permeable structure regulating ion and nutrient transport into the brain. It represents a filter between the CNS and the blood, limiting the free flow of physiological molecules between the bloodstream and parenchyma. Specialized endothelial cells (kept strictly joined by tight junctions), which line cerebral blood vessels, are surrounded by a basal lamina and astrocyte end-foot processes to form and maintain the BBB [103]. Other barriers are constituted by the choroid plexus epithelium between blood and the ventricular CSF and the arachnoid epithelium between blood and the subarachnoid CSF [103].

Alterations of the BBB integrity can facilitate the passage of potentially harmful substances into the brain, with possible pathological consequences. During CM, vascular dysfunction may cause severe damage to the BBB, enhanced by the inflammatory cascade mentioned above [102].

In many neurological diseases, disruption of the BBB is mediated by the MMPs [reviewed in 96]. In fact, MMPs derived from infiltrating leukocytes or from cells of the CNS cleave matrix proteins, which are essential for the maintenance of BBB integrity and for neuronal survival [104]. In fact, intraparenchymal injection of MMPs causes breakdown of the BBB and the subsequent leakage of capillaries. Furthermore, MMP inhibitors, such as tissue inhibitor metalloproteinase (TIMP)-2, reduce extracellular matrix proteolysis induced by type IV collagenase and protect the BBB [105]. This barrier does not restrict leukocyte diapedesis; in fact, a small number of activated lymphocytes can access the CNS with the aim of developing an immunosurveillance to control possible infectious agents [106].

Both MMPs and TIMPs can play a relevant role in malaria pathogenesis with at least two mechanisms: (i) degradation of BBB substrates and (ii) functioning as effectors and regulators of the |immune response.

The results obtained in malaria patients are contradictory. In fact, while in Kenyan children affected by acute malaria, by means of a genome-wide analysis of expression, an upregulated MMP-9 mRNA expression in blood cells was observed, accompanied by neutrophilia [107]. In Gabonese children affected by either uncomplicated or severe malaria, MMP-9 serum levels were unchanged compared to healthy controls, with TIMP-2 levels even lower. On the contrary, TIMP-1 is associated with signs and symptoms of severe malaria, and MMP-8 levels are elevated in patients with severe or uncomplicated *P. falciparum* malaria [108]. In the light of these results, it may be argued that TIMP-1 and MMP-8 may predict the severity of malaria. TIMP-1 may prevent further MMP-induced damage by counterbalancing the activity of MMP- 9 and, to a lesser extent, MMP-8 activity. 

Data derived from experimental studies are accumulating, which show unequivocally that MMPs and TIMPs, as well as the TNF-α converting enzyme, play a crucial role in malaria pathogenesis [96].

As in other inflammatory conditions involving the brain, such as lipopolysaccharide (LPS)-injured brain or multiple sclerosis [109,110], MMP-9 might also play an important role in CM pathogenesis, increasing BBB permeability and infiltration of leukocytes. In particular, this gelatinase seems to be induced by the hemozoin, a catabolic product of hemoglobin, at the endothelial cell level, as shown in *in vitro* studies. Furthermore, this parasite-derived product increases the protein expression of MMP-1, MMP-3 and also of TIMP-2 [111].

In patients infected by *P. falciparum*, analysis of post-mortem brain samples showed that MMP-1 accumulates in astrocytes of the BBB and in macrophages/microglial cells, which are present in Dürck's granulomas [112], represented by microglial-astroglial nodules surrounding the damaged vessels (see Figure 2 from Deininger *et al*. [113]). 

**Figure 2:**
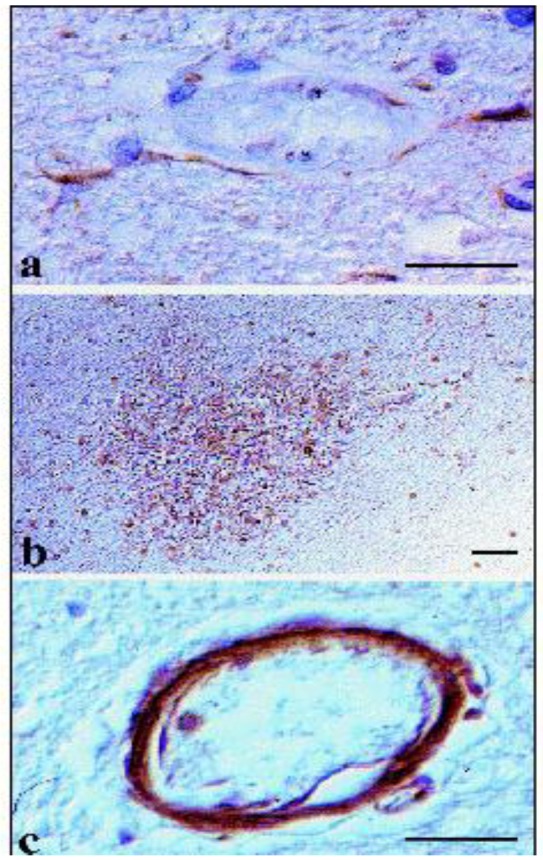
Some aspects of pathogenesis of cerebral malaria. In brains of patients who died with cerebral malaria, TGF-beta1 immunoreactivity (brown color) was found in astrocytes that form the blood-brain barrier around cerebral capillaries, characterized by deposition of malarial pigment and sequestration (**a**). TGF-beta2 (brown color) immunoreactivity was found in macrophages/microglial cells in Dürck´s granulomas and in glioses of ring hemorrhages (**b**). TGF-beta3 immunoreactivity (brown color) was found in endothelial and smooth muscle cells in capillaries with deposition of malarial pigment and sequestration (**c**). All slices were counterstained with hematoxylin eosin. Bars = 25 μm. (From [113], with permission.)

From all these data, we may argue that in malaria caused by *P. falciparum*, especially if complicated by CM, the balance between MMP and TIMP expression patterns is altered in favor of the former. This imbalance is responsible for the dramatic disruption of the BBB, which occurs during CM. However, the definitive demonstration of the role played by the MMPs in disrupting the BBB during CM is still not available, and further research on this issue is needed [96].

#### 4.1.2. African Trypanosomosis

African Trypanosomosis is caused by protozoa of the genus *Trypanosoma* (*T. brucei*), which are responsible for one of the most important parasitic infections in Sub-Saharan Africa. These trypanosomes cause human sleeping sickness (human African trypanosomiasis, HAT) in man and a cattle disease, called Nagana. The disease evolves from a first hemolymphatic stage to a second meningo-encephalitic stage, after parasites cross the BBB and invade the CNS [114]. 

After injection by the vectors (tsetse fly), the parasites (at the stage of metacyclic trypomastigotes) change morphology and behavior in the circulation; therefore, they acquire the ability to cross the BBB [115]. At this phase of infection, the so-called meningoencephalitic stage occurs. After passing through the highly vascularized epithelium of the choroid plexus, the parasites enter into the CSF, and later, they arrive to the brain parenchyma. At that time, an induced expression of host endothelial receptors, e.g., ICAM-1, is observed. Accumulation of parasites in the brain, followed by the recruitment of leukocytes and activation of astrocytes and microglia, cause chronic encephalopathy, which may be fatal, if not treated [116].

The disruption of BBB during African trypanosomosis is controversial; it was observed, for example [117], that the parasite can pass the barrier by means of a cysteine proteinase, in part by generating Ca^2+^ activation signals. However, the traversal of leukocytes and parasites through the basal lamina into the brain at second stages of HAT may have common mechanisms involving certainly the MMPs.

MMP-2 and MMP-9 were significantly increased; results correlated with the presence of parasites and leukocytes in the CSF of *T. b. gambiense*-infected patients with second-stage HAT.

It was therefore highlighted that MMP-9, along with ICAM-1, are valuable staging markers for *T. b. gambiense* HAT and that, alone or in combination, MMPs, as well as ICAMs, can identify the meningo-encephalitic stage of HAT [118]. It has been observed that MMP-2 and MMP-9 create a localized temporary opening of the glia limitans (which is a part of the BBB) by selective cleavage of the β-dystroglycan subunit anchoring the astrocyte end-feet to the parenchymal membrane [119]. In this way, the gelatinases MMP-2 and MMP-9 let leukocyte penetrate the outer parenchymal basement membrane into the brain parenchyma.

In the brain of *T. b. brucei*-infected mice, a massive increase in parasite and leukocyte numbers was accompanied by a significantly induced mRNA expression of MMP-3, MMP-8 and MMP-12, at thirty days post-infection, whereas levels of MMP-1b, -2, -7, -9, -11, -13, -14 and -19 and TIMP-1 and -2 mRNA were unaltered and MMP-10 was undetectable [120]. 

We may therefore conclude that, also in trypanosomosis, like in cerebral malaria, increased MMP expression levels are responsible for the passage of the parasites, as well as leukocytes, through the BBB into the brain, where they cause cerebral pathology. 

#### 4.1.3. Toxoplasmosis

*Toxoplasma gondii* is an obligate intracellular parasite capable of infecting virtually any warm-blooded animal [121]. In humans, *Toxoplasma* infections are widespread and can lead to severe disease, *i.e*., toxoplasmic encephalitis in individuals with an immature or suppressed immune system [122].

In murine *Toxoplasma* encephalitis, CD4+ and CD8+T cells are mainly recruited to the brain, where they are crucial in preventing the reactivation of latent infections [123]. This process is mediated by IFN-γ; this cytokine, in fact, stimulates anti-parasitic effector mechanisms, for example, of macrophages and regulates chemokine expression and leukocyte recruitment [124]. Different molecules involved in the immune and inflammatory response during toxoplasmosis, such as IL-1, IL-23, TNF-α and COX-2, increase the MMP production in the brain, reviewed by Clark and colleagues [125].

In the brain tissue of *T. gondii*-infected mice, an increase in CD4+ and CD8+ T cells producing both MMP-8 and MMP-10 has been recently shown [125]. Furthermore, TIMP-1 is expressed in invading T-cells and CNS-resident astrocytes during infection. In wild-type mice, changes in tissue morphology and signs of astrocyte activation occur, contrary to what happens in infected TIMP-1 KO mice, where an increase in CD4+ T cells along with a significantly reduced parasite burden in the brain was observed, differently from the peripheral amount of parasites. This is not accompanied by any substantial pathology in the brain, as shown by histology, which suggests less focal immune clusters and decreased astrocyte activation.

It may be argued that upregulation of TIMP-1 during infection may inhibit the pathogen clearance by limiting lymphocyte penetration into the CNS, driven by MMPs. 

The resulting inhibition of MMPs by an increased expression of TIMP-1 may represent an evasion mechanism by the parasite, with the aim to limit the arrival of immune cells or a host response to downregulate immune-mediated pathology. 

In infected TIMP-1 KO mice, in fact, brain inflammation occurred with a reduced or even absent perivascular accumulation, but with a higher number of CD4+ T-cells infiltrating the brain parenchyma, probably reflecting a lack of control of the degradation processes of the basal lamina, played by MMP. All these data suggest that MMPs are beneficial in facilitating the parasite clearance from the brain.

### 4.2. MMPs and Helminth Infections

#### 4.2.1. Neurocysticercosis

Neurocysticercosis (NCC) is the CNS infection caused by the larva of *Taenia solium* tapeworm, acquired after parasite egg ingestion (NCC without taeniasis) or raw or poorly cooked pork consumption (NCC with taeniasis) [126]. It is considered the most common cause of acquired epilepsy at the global level.

The clinical picture is pleomorphic, but active seizure is the most frequent manifestation. The disease is slowly progressive, and multiple factors play a role in the severity of the symptoms; one of them is represented by the degree of inflammatory reaction in the host brain. It is also possible, however, that individuals with NCC in many cases could remain asymptomatic, and the exact reasons largely remain unexplained [127]. 

**Figure 3:**
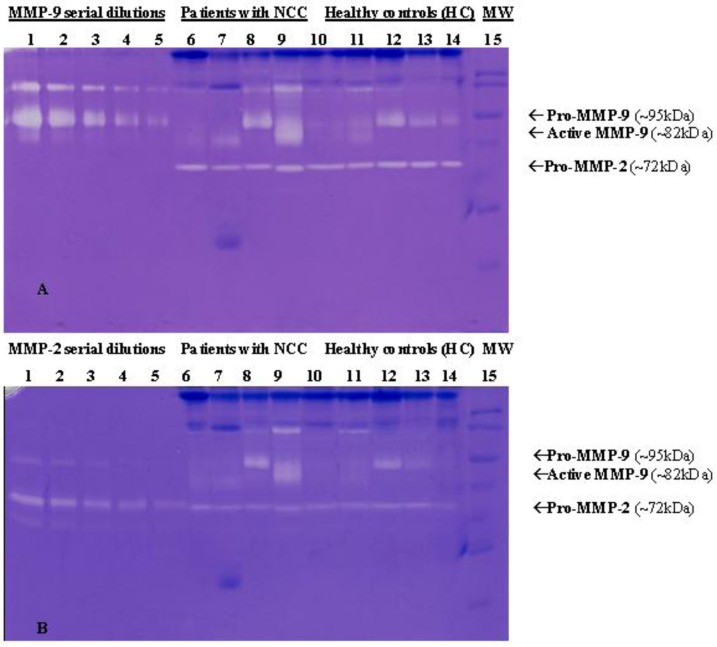
Example of gelatin zymography for detection of MMP-9 and MMP-2 activities in serum samples of patients with neurocysticercosis (NCC) and healthy controls (HC). Human Pro-MMP-9 (~ 95 kDa) and Pro-MMP-2 (~ 66 kDa) standards were used as positive controls (Calbiochem Co.). Lanes 1–5 in gel A correspond to serial dilution of recombinant pro-MMP-9; lanes 1–5 in gel B correspond to serial dilutions of recombinant MMP-2. For both gel A and gel B, lanes 6–10 are samples of infected individuals (line 6 and line 10 correspond to individuals with asymptomatic neurocysticercosis); lanes 11–14 are control groups of non-infected individuals; in lane 15, a molecular weight marker (MW) (Bio-Rad, U.S.A.) was loaded.

Studies in experimental animals have shown that the MMP expression plays a crucial role in the differential breakdown of the BBB, from which infiltration of blood leukocytes and, consequently, the production of inflammatory cytokines depend. In this model of NCC, inflammatory infiltrates contain different populations of immune cells, and different MMPs are involved in the mechanisms of cleavage of cytokine, chemokine and adhesion molecules, by which the immune response can be activated [128]. In patients with NCC, serum levels (evaluated by ELISA) and enzymatic activities (evaluated by zymography) of MMP-2 and MMP-9 resulted in association with symptomatic manifestations of the disease. Figure 3 shows an example of gel zymography, where NCC serum samples were loaded.

Mean serum MMP-2 levels were higher both in asymptomatic and symptomatic NCC cases compared to healthy controls; however, there was no difference in the levels of MMP-2 in symptomatic and asymptomatic NCC patients. 

**Figure 4:**
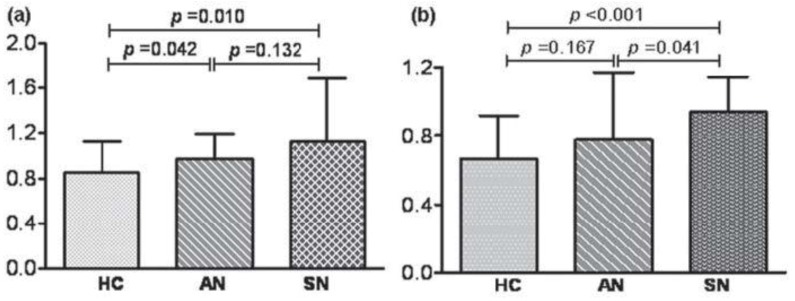
Zymography analysis of matrix metalloproteinase (MMP) activities in sera of healthy controls (HC), asymptomatic neurocysticercosis (AN) and symptomatic neurocysticercosis (SN). (**a**) MMP-2; (**b**) MMP-9. From [129], with permission.

On the contrary, MMP-9 serum levels were significantly higher in symptomatic NCC patients than in asymptomatic NCC cases or healthy controls (Figure 4). Levels of both MMPs positively correlated with symptomatic (presence of seizures) NCC [129].

Recent studies have shown that higher levels of MMP-9 correlated with epilepsy [130,131]. In consideration of that, symptomatic NCC patients might underlie the seizures, because of increased levels of MMP-9.

#### 4.2.2. Infections with Nematodes

Metalloproteinases have been identified in a variety of nematodes, such as *Brugia malayi*, *Toxocara canis*, *Strongyloides stercoralis*, *Nippostrongylus brasiliensis*, *Dirofilaria immitis*, *Trichuris suis*, *Ancylostoma caninum*, *Caenorhabditis elegans* and *Gnathostoma spinigerum* (reviewed in Tsai *et al.*, 2008) [132]. However, one of the most studied model *in vivo* as regards MMPs involvement in the pathogenesis of nematode-induced cerebral infections is certainly represented by *Angiostrongylus cantonensis*, a nematode parasite responsible for the most common eosinophilic meningitis in the Pacific Islands and Southeast Asia [132].

This rat lung worm has an obligatory intracerebral migration in its hosts [133]. When humans and mice (nonpermissive hosts) are infected with this parasite, the worms migrate to the brain, where they develop into young adults, failing to reach maturity within the heart and lungs [133]. *A. cantonensis* is a neurotropic nematode that requires the CNS of mammalian hosts for its growth [134].

The infective third-stage larvae orally infect the final host and are carried in the blood to the CNS, where they molt twice to become immature adults and enter the subarachnoid space. In a permissive host (rodents), immature adults migrate from the brain to the lungs [133].

However, in non-permissive hosts, the immature adults remain in the CNS of the host, and this infection is the main cause of eosinophilic meningitis and eosinophilic meningoencephalitis [135]. Most of information on this parasitic infection derives from experimental studies in rodents. Mice infected with *A. cantonensis* undergo eosinophilic meningitis, which peaks at around three weeks, when CSF eosinophilia reaches a peak [136,137]. It was also shown that the MMP-9 was detected in CSF at day 10 post-inoculation (PI) and reached a high level from days 15 to 25 PI. This increased level was not due to parasitic production of MMP-9, since excretory/secretory (E/S) antigen from *A. cantonensis* adult worms lacked the enzyme activity. However, analysis of the extracts and E/S products of the parasite larval and adult stages on gelatin substrate zymography demonstrated the presence of distinct gelatinolytic enzymes. In worm extracts, it was found that the metalloproteinases at different molecular weights: in L1 23 kDa, in L3 66, 42 and 30 kDa, in young adult worms, 72 and 94 kDa, and in adult worm, again 72 and 94 kDa. On the contrary, in E/S products, the L1 revealed one low (42 kDa) and two high (105 and 94 kDa) molecular weight gelatinolytic bands. The L3 revealed three low (66, 50 and 30 kDa) and one high (105 kDa) molecular weight proteolytic bands. By using specific inhibitors, the nature of metalloproteinases was confirmed for the 105 and 94 proteolytic bands of the L1 and for the 50 and 30 kDa proteolytic bands of the L3 larvae. These metalloproteinases secreted in the infective larvae might be associated with the parasite dissemination or pathogenesis [138].

By using Immunohistochemistry, the MMP-9 localized within eosinophils and macrophages, present in the subarachnoid space of experimentally infected mice. 

These data suggest that infiltrating leukocytes are important sources of MMP-9 in this parasitic meningitis [139]. In particular, by immunogold electron microscopy, it was shown that in eosinophils, MMP-9 was mostly localized in the 'small' granules in the cytoplasm and along the cell membrane and not in the crystalloid-containing secretory granules observed, suggesting that the enzyme is synthesized and/or stored in the small granules of the eosinophils, to be released into the subarachnoid space of the host's brain by secretion or cell rupture [140]. MMP-9 was also found within the endothelial cells lining the vascular spaces of the brain [141].

The increased MMP-9 activity was significantly associated with the rapid increase of CSF eosinophils and the inflammatory reaction of the subarachnoid space. Contrary to what happens for MMP-9, MMP-2 activity did not change during infection [139].

During experimental infection in mice, Purkinje cells in the cerebellum become small and irregular in shape, with degenerative atrophy or partial loss; furthermore, enlarged vacuolar structures and swollen mitochondria within the cytoplasm are visible at electron microscopy. The MMP-9 mRNA expression, which is absent in normal Purkinje cells, precedes the degeneration process. Then, MMP-9 protein level and enzyme activity increased when the Purkinje cells were degenerated. Furthermore, MMP-9 was localized within degenerative Purkinje cells. 

When a specific MMP inhibitor was used, MMP-9 enzyme activity decreased by 41.6%. In addition, the number of degenerated Purkinje cells was reduced, as well [142]. 

Proteolysis depends on the balance between the proteinases and their inhibitors. MMP-9 and its specific inhibitors, TIMPs, contribute to eosinophilic inflammatory reaction in the subarachnoid space of the *A. cantonensis*-infected mice. If MMP-9 levels increase during infection, those of TIMP-1 did not change, remaining at basal levels at all time points. Immunohistochemistry demonstrated that also TIMP-1 is localized in eosinophils and macrophages infiltrating the CNS tissue. These results show that MMP-9/TIMP-1 imbalance in angiostrongyloidosis may be associated with eosinophilic meningitis [143].

As already stated, blood–central nervous system barrier breakdown is an important pathophysiological event occurring in meningitis, which is the result of extravasation of leucocytes into subarachnoid space. Two enzymatic systems are essentially responsible for this disruption, the plasminogen activators (PAs) and MMPs [143]. In mice experimentally infected with *A. cantonensis,* it was shown that during eosinophilic meningitis the activities of tissue-type PA (tPA), urokinase-type activator (uPA) and MMP-9 in cerebrospinal fluid (CSF) were significantly increased, compared to uninfected animals. Furthermore, eosinophilia present in the CSF significantly correlated with tPA, uPA and MMP-9 activities, as well as with albumin, content in the fluid. In addition, when infected mice were treated with a specific MMP blocker, MMP-9 activity and total protein concentrations declined significantly, suggesting that the PAs and MMP-9 proteolytic cascade may be involved in blood–CNS barrier disruption, during eosinophilic meningitis [144].

CSF levels of MMP-2, MMP-9 and TIMP-1 resulted in significantly higher levels in patients infected by *A. cantonensis* in Taiwan, suffering eosinophilic meningitis, compared with healthy controls. On the contrary, TIMP-4 levels were significantly lower in the same patients. In contrast to MMP-2, proteolytic activity of MMP-9 detected by gelatin zymography was only observed in patients with eosinophilic meningitis. Higher MMP-9 levels were found in the CSF of patients with eosinophilic meningitis, contrary to what happens for MMP-2. CSF MMP-9 increases in patients in parallel with CSF leukocyte counts and CSF/serum albumin ratio (QAlb) values. During recovery from eosinophilic meningitis, after treatment with mebendazole and dexamethasone, a gradual decrease in levels of MMP-9 (decreased more than 50% in six patients two weeks after treatment), QAlb and TIMP-1, as well as an increase in those of TIMP-4, were observed. TIMP-4 levels were lower during the acute phase of infection, persisting as such even when MMP-9 and TIMP-1 have already decreased, until eosinophil meningitis was not definitely recovered, suggesting that TIMP-4 plays a crucial role in the proteolytic balance of BBB damage, in these patients. These results confirmed what was already known from studies in experimental infection models. 

In the CSF of patients, activity of MMP-9 was not completely inhibited, because of the simultaneous decrease of TIMP-4, with the resulting BBB dysfunction, as shown by the higher CSF/serum albumin ratio (QAlb) values observed in these patients. Modification of the cytokine milieu has a major impact on modulation of TIMP expression and may be responsible for changes in the levels of these proteins (MMPs/TIMPs) in eosinophilic meningitis [132].

Also, MMP-12 and its substrate, elastin, participate in the inflammatory response. MMP-12/TIMP-1 ratio was significantly increased in the CSF of *A. cantonensis*-infected mice from day 10 p.i. (post-infection) and reached high levels on days 20 and 25 p.i. The production of MMP-12 resulted correlated with several parameters, such as elastin degradation, eosinophil count, blood–CSF barrier permeability and pathological changes in the subarachnoid space.

MMP-12 might be involved in elastin degradation in the meningeal vessel of the subarachnoid space. After treatment with both albendazole (an antihelmintic) and doxycycline (used in this study as a non-selective MMP inhibitor), a significant reduction of the levels of MMP-12, elastin and Evans blue accumulation in the CSF in mice with meningitis was observed, suggesting that MMP-12 contributes to elastin degradation, which is reduced by the action of doxycycline on the inflammatory reaction mediated by MMP-12 [145].

## 5. Concluding Remarks

A common aspect of parasitic infections (caused by *Plasmodium*, African *Trypanosoma*, *T. gondii*, *T. solium*, *A. cantonensis*), which may involve the CNS, is represented by increased levels of several MMPs, which are induced either directly or indirectly by regulating cytokine levels; therefore, with an imbalance between such enzymes and TIMPs.

A summary of MMP and TIMP levels in the different parasite infections involving the CNS is shown in Table 2.

**Table 2:** Summary of MMP and tissue inhibitor metalloproteinase (TIMP) modifications during parasitic infections of the Central Nervous System (CNS).

Parasitic infection	MODIFICATION OF MMP levels	MOFIFICATION OF TIMP levels	Refs.
Cerebral malaria	MMP-9 levels increased or unchanged	TIMP-2 level decreased	[107,108]
	MMP-8 level increased [108]	TIMP-1 level increased	[108]
	MMP-1 accumulation in CNS [113]		[113]
African trypanosomosis	MMP-2 and MMP-9 levels increased	TIMP-1 and TIMP-2 levels unchanged	[117,120]
Cerebral toxoplasmosis	MMP-8 and MMP-10 produced by CD4+ and CD8+ T cells	Expression of TIMP-1 in the CNS	[125]
Neurocysticercosis	MMP-2 and MMP-9 levels increate in symptomatic patients [129]		[129]
Angiostrongyloidosis	MMP-9 accumulation in inflammatory cells invading the CNS in experimental infection [139]		[139]
		TIMP-1 level increased in patients	
	MMP-2 and MMP-9 levels increased in patient	TIMP-4 level decreased	[132]

The final result of this phenomenon is represented by various forms of (meningo)encephalitis, which follow the migration of different leukocyte populations and/or parasites across the BBB. In all these examples of cerebral parasitic diseases, the activity of MMPs is crucial for either the migration of inflammatory cells and parasites and the disruption of BBB integrity. 

For that reason, MMPs might represent suitable therapeutic targets to prevent the BBB disruption, not only in protozoan [96], but also in helminth infections. 
